# Trends and geographic variability in gender inequalities in child mortality and stunting in India, 2006–2016

**DOI:** 10.1111/mcn.13179

**Published:** 2021-03-14

**Authors:** Harold Alderman, Phuong Hong Nguyen, Lan Mai Tran, Purnima Menon

**Affiliations:** ^1^ Poverty, Health and Nutrition Division International Food Policy Research Institute (IFPRI) Washington District of Columbia USA; ^2^ FHI Solution Hanoi Vietnam

**Keywords:** boys, girls, India, inequality, mortality, stunting

## Abstract

Gender disparities in child undernutrition and mortality in India have been a topic of interest for a long time, but little is known on trends or geographic variability in recent periods. We examined the degree to which historic patterns in gender disparities in child undernutrition and mortality in India have persisted given recent progress in health and nutrition. Using two nationally representative datasets from India between 2006 and 2016, we estimated mortality rates and stunting by gender and by birth order among children under 5 years old. We then tested for differences between boys and girls within each survey round for both national and state levels using bootstrapped standard errors, controlling for cluster and sampling weights. We found striking progress in child mortality and stunting in India between 2006 and 2016 for both boys and girls. Boys were more likely to die than girls during the first year of life. Girls had a higher risk of mortality between age 1 and 5 years than boys in 2006, but the improvements in survival eliminated this gender gap in 2016. For stunting, we found no gender difference in 2006, but girls had higher height‐for‐age *Z*‐scores (HAZ) and lower stunting than boys in 2016. Trends in gender gaps in mortality and stunting vary substantially by birth order and between states. Our findings indicate that improvements in mortality and nutritional status among girls have started to close gender disparities. Policy efforts to close gaps must stay the course in states that have made progress and be accelerated in states where disparities are still prominent.

**Key messages**

- Gender disparities in child undernutrition and mortality in India have been a topic of interest for a long time. However, previous studies on this topic generally reporting a disparity favouring boys only used data before 2006.
- This study offers an in‐depth analysis to ascertain the degree to which historic patterns in gender disparities in child undernutrition and mortality in India have persisted given recent progress in health and nutrition. Our findings indicate that improvements in mortality and nutritional status among girls have close gender disparities.
- Staying the course to ensure that economic, social and health policies remain in place to support further improvements in health and nutrition will be important for India's girls; this is especially important in the context of massive disruptions like the COVID‐19 pandemic.

## 1 INTRODUCTION

There has been much discussion about pervasive gender disparity in mortality in Asia in general and India specifically. This pattern was brought to the fore with a dramatically titled paper ‘More than 100 Million Women are Missing’ (Sen, 1990). Few researchers doubt the basic population gender disparity in India, but there is debate as to the ages at which it occurs and the degree to which it is evolving. For example, a study using survey data from the 1990s claims that differences in under 5 mortality are sufficient to explain the population gender disparity in India (Oster, 2009), whereas a later study in 2010 employs World Health Organization (WHO) population data and concludes that the missing women reflect adult mortality (Anderson & Ray, 2010). There is also some debate as to whether gender disparity in mortality has increased or decreased since the 1990s. Whereas some studies show evidence of an increase in gender disparity in mortality in India at the end of the 20th century (Das Gupta & Mari Bhat, 1997), others see a global decrease in the number of missing women as a percentage of the population with India experiencing a smaller decline than its neighbours yet having movement in the same direction (Klasen & Wink, 2002).

Researchers have also pointed out that gender patterns in child mortality differ substantially by age; neonatal mortality has different underlying causes as well as different outcomes than mortality in later childhood (Kim et al., 2020; Million Death Study et al., 2010). Preventable deaths in the former largely reflect maternal nutrition, antenatal care and unsafe delivery and thus are less mediated by gender‐specific patterns in childcare (Million Death Study et al., 2010). Such childcare patterns include, for example, the fact that girls in India may be breastfed for shorter periods than boys (Jayachandran & Kuziemko, 2011) or have longer delays in seeking care when the child is ill (Malhotra & Upadhyay, 2013; Mishra et al., 2004).

Given that malnutrition is associated with a large share of mortality in India (India State‐Level Disease Burden Initiative Malnutrition, 2019), evidence on gender patterns in undernutrition is often presented along with studies of mortality (Barcellos et al., 2014; Oster, 2009). India is known as a country with son preferences (Hvistendahl, 2011) where boys have historically had an advantage in height and weight and lower undernutrition prevalence relative to girls (Barcellos et al., 2014). This, again, may be a reflection of differences in childcare, either in terms of time or in terms of expenditures. These investments vary over the lifecycle and depend on the child's age as well as the outcomes of earlier decisions if they survive. These investments can either reinforce or counteract health disparities within a household (Alderman et al., 2017). Thus, just as patterns in neonatal mortality may differ from mortality in later childhood years, gendered patterns in nutritional outcomes need not necessarily track disparities in mortality (Alderman et al., 2017).

Moreover, access to health infrastructure and, subsequently, health outcomes are changing rapidly in India. Between 2006 and 2016, child mortality and stunting reduced considerably in India (from 74 to 50 per 1000 birth for under 5 mortality rates [U5MR] and from 48% to 38% for stunting). During this period, the country rolled out ambitious health programmes through the National Rural Health Mission (Government of India, 2009, 2014) and scaled up nutrition services under the Integrated Child Development Services (ICDS) scheme (Chakrabarti et al., 2019). The interventions delivered through these programmes focused on addressing child mortality and undernutrition, notably through efforts to scale‐up immunization, micronutrient supplementation and deworming, food supplementation, growth monitoring and management of severely acute malnourished children (Avula et al., 2013; Vir et al., 2013). Research has shown the impact of this improved intervention coverage on reducing undernutrition and mortality at national and subnational levels (Alderman et al., 2019). A recent study has also examined the trends in child mortality in India between 2000 and 2017 and reported substantial variations between the states and districts in the magnitude and rate of decline in mortality but did not analyse gender inequality (India State‐Level Disease Burden Initiative Child Mortality, 2020).

Previous studies that attempted to explain sex differentials in child mortality (Arokiasamy, 2004; Gupta, 1987; Kuntla et al., 2014) or malnutrition in India and its associated factors (Corsi et al., 2015; Pillai & Ortiz‐Rodriguez, 2015) used data before 2006. To our knowledge, only one recent study has explored the gender disparity in the changes of mortality using updated data in the last decade (Karlsson et al., 2019). While the current study reaches core conclusions similar to that recent report, it also includes an exploration of the variability in these trends by state as well as evidence as to how trends in mortality compare to those in nutrition. This exploration is important because both health investments and social conditions that contribute gender differentials have not remained constant either in time or across states in India.

Specifically, this paper updates analyses on trends in mortality by gender in India, disaggregating neonatal mortality rates (NMR) from infant mortality rates (IMR) and U5MR. We compare rates in Indian states in 2005–2006 with corresponding rates in 2015–2016. We also investigate trends in gender disparity in stunting among children under less than 2 years old as well as children 2 to 5 years old.

## 2 METHODS

### 2.1 Data sources

This paper utilized individual data from the third and fourth rounds of the National Family Health Survey (NFHS‐32006 and NFHS‐42016). All data are in public domain and can be downloaded from the Demographic and Health Surveys (DHS) website (https://www.dhsprogram.com/) after obtaining permission from the DHS programme. Detailed survey sampling procedures and questionnaires are available in the final reports of NFHS‐3 (International Institute for Population Studies [IIPS], 2008) and NFHS‐4 (IIPS, 2018). Briefly, both these cross‐sectional surveys follow a systematic, multistage stratified sampling design used in DHS in many other countries. The first stage involved selection of primary sampling units (i.e. villages in rural areas and Census Enumeration Blocks in urban areas) using probability proportional to population size. The second stage involved the random selection of 22 households from each primary sampling unit. The response rate was high for both survey rounds (94.5% response rate among women in NFHS‐3 and 96.7% in NFHS‐4). We use data from all women aged 15–49 years and their children under 5 years of age in selected households (*n* = 51 555 for 2006 and 259 627 for 2016). Whereas NFHS‐3 is representative at the state level, NFHS‐4 is representative at both the state and district levels.

### 2.2 Variables

#### 2.2.1 Mortality rates

Childhood mortality rates were estimated for Indian states, using mothers' reports on the date of birth of each of their children, their survival status and the dates of death or ages at death of deceased children. We applied the direct method as guided in the DHS reports, using the Stata Version 16.0 package ‘syncmrates’, which calculates age‐specific mortality rates using the synthetic cohort probability method (Rutstein & Rojas, 2003). This approach allows full use of the most recent data and is also specific for time periods. The reference period of these estimates is 5 years prior to the survey date and, thus, avoids overlap of cohorts.

IMR and U5MR are defined as the number of deaths per 1000 live births by the age of 1 and 5 years, respectively. Mortality rates for children aged 1–5 years are the differences between U5MR and IMR and are termed child mortality rates (CMR) in the analysis. NMR reflect births in the first 28 days of life, whereas postneonatal mortality (PMR) is the number of deaths that occur between 28 and 365 days per 1000 live births.

#### 2.2.2 Child undernutrition

Child anthropometry was collected by trained and standardized field staff using standard methods, as described in NFHS‐3 and NFHS‐4 reports (IIPS, 2008, 2018). Recumbent length/height of the children was measured by Seca 417 infantometer for children below 2 years and Seca 213 stadiometer for children 2–5 years. Length/height was then converted into height‐for‐age *Z*‐scores (HAZ), according to 2006 WHO child growth standards. Stunting is defined as < −2 *Z*‐score of HAZ.

### 2.3 Statistical analyses

We estimated mortality rates by gender and birth order at the national level and also calculated mortality rates by gender at the state level. For HAZ and stunting, we estimated weighted means and plotted the distributions of HAZ and stunting against child age and sex using a local polynomial smoother. Given the difference in growth patterns by age groups, we report various descriptive statistics of HAZ scores and stunting prevalence for each round of survey for children <2 and 2–5 years separately. For child mortality, we applied the ‘syncmrates’ command together with the *T*‐test option to examine the difference in mortality rates by child sex. This technique calculated bootstrapped standard errors and confidence intervals for the mortality rates and allows all Stata bootstrap options. For child HAZ and stunting, we tested for differences between boys and girls within each survey year using regression analyses with ‘svy’ command to control for the cluster sampling design and sampling weights used in the survey. Statistical significance was considered at three levels: *p* < 0.05, *p* < 0.01 and *p* < 0.001.

## 3 RESULTS

The mean age of children was 29.7 months in both survey rounds with ~40% of children aged <2 years and 60% aged 2–5 years. The gender ratio was 108.2 boys per 100 girls in 2006 and 108.5 in 2016 (Table 1). Birth order in both surveys was distributed similarly among boys and girls. The proportion of girls and boys who were ≥ 4th born in 2006 was higher than that in 2016 (23% vs. 16%) reflecting changes in fertility.

**TABLE 1:** Study sample by gender and birth order in India, 2006–2016

	2006				2016			
	Total	Girls	Boys	Gender ratio	Total	Girls	Boys	Gender ratio
	n	%	%	Boys/girls	n	%	%	Boys/girls
First child	16 567	48.4	51.6	105	96 212	48.1	51.9	107
Second child	14 409	48.2	51.8	109	79 670	48.0	52.0	108
Third child	8318	46.8	53.2	115	41 607	46.5	53.5	112
Fourth child and above	12 261	47.7	52.3	108	42 138	47.8	52.2	108
Total	51 555	24 756	26 799	108.2	259 627	124 525	135 102	108.5

**FIGURE 1:**
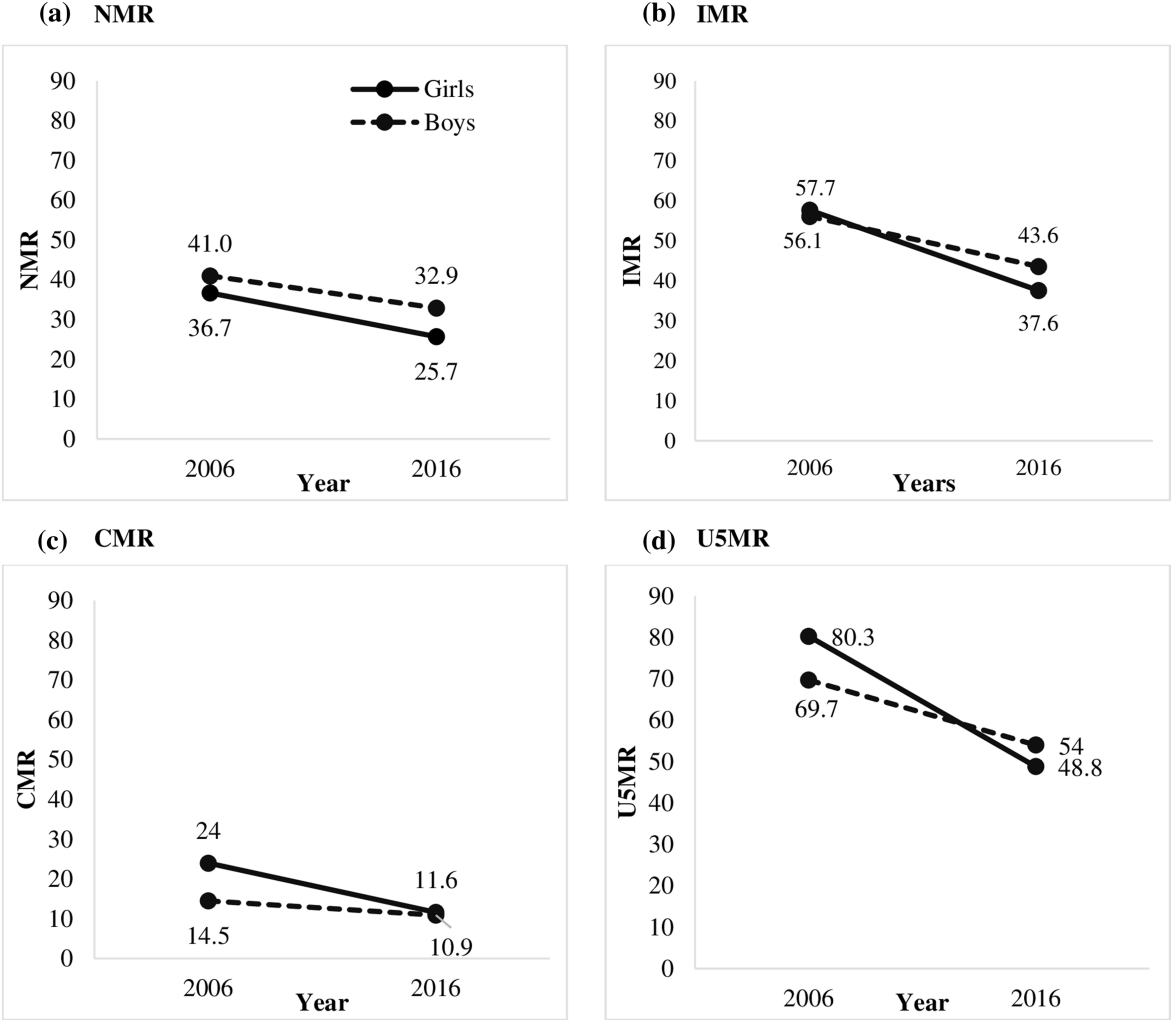
Child mortality rate by gender and survey year in India, 2006–2016. (a) Neonatal mortality rates (NMR). (b) Infant mortality rates (IMR). (c) Child mortality rates (CMR). (d) Under 5 mortality rates (U5MR)

Boys are more likely to die than girls in the first 28 days after birth (Figure 1). Although overall NMR declined appreciably between 2006 and 2016, the average gap between the NMR of boys and that of girls widened from four deaths per 1000 live births in 2006 to seven deaths per 1000 a decade later. The higher mortality rate among boys compared with girls is consistent with the patterns of previous surveys (57 vs. 48 in 1992 and 51 vs. 45 in 1999) (Figure S1). However, there were appreciable differences across states; in Uttar Pradesh, Bihar and Madhya Pradesh, the decline in NMR for boys was far smaller than the decline in NMR for girls (Table S1). This contributed to the increase in the mortality disparity in that age bracket. Given that most deaths in the first year of life occur in the first month, the gender disparity in IMR tracks that in NMR (Figure 1 and Table S2).

In 2006, girls who survived their first year had a higher risk of subsequent mortality before their fifth birthday than did boys (Figure 1). This was mainly driven by wide disparities in CMR in Uttar Pradesh as well as Rajasthan (Table S3). This pattern is also observed in the NFHS‐1 and NFHS‐2 (CMR for boys vs. girls was 29.4 vs. 42.1 in 1992 and 24.8 vs. 36.6 in 1999) (Figure S1). By 2016, these disparities had largely disappeared in Rajasthan and Uttar Pradesh as well as the nation overall. U5MR reduced substantially in the last two decades (Figure S1), and the gender differences in U5MR (Figure 1 and Table S4) are similar as those for CMR. Whereas girls in the north‐west states of Jammu and Kashmir, Punjab and Rajasthan had higher U5MR in 2006, the latter significantly so, the differences declined by 2016 as it had in Uttar Pradesh.

Mortality does not increase monotonically by birth order (Table 2). First‐born children, whether girls or boys, are at greater risk of NMR or IMR than second‐ or third‐born children. However, at higher birth orders, the risk of early mortality increases and did so more for girls in 2006. In both 2006 and 2016, the NMR for boys who were a first‐born child was higher than for girls, but this difference largely disappeared for fourth‐born children. Indeed, the difference in point estimates reversed sign in 2006 for NMR and IMR. CMR increases monotonically—and sharply—for higher birth order girls compared with those born first in both surveys, although the absolute rates are lower at any birth order in 2016 than in 2006. The role of birth order is also sharp for U5MR. In 2016, second‐ or third‐born boys have nearly the same risk of dying as first‐born boys, whereas those later in the birth order have an elevated risk of CMR and U5MR compared with other boys.

**TABLE 2:** Child mortality rate by gender and birth order in India, 2006–2016

	2006			2016		
	Girls	Boys	Gaps	Girls	Boys	Gaps
**NMR among child <1 month**						
First child	41.4	53.5	12.1*	26.7	36.7	10.0***
Second child	29.5	34.1	4.6	19.2	26.2	7.0***
Third child	26.4	28.5	2.2	26.3	29.6	3.3
Fourth child and above	44.7	41.6	−3.1	36.8	41.6	4.8*
All children <1 month	36.7	41.0	4.3	25.7	32.9	7.3***
**IMR among child <1 year**						
First child	59.5	69.5	10.0	35.7	46.5	10.8***
Second child	47.2	45.8	−1.4	29.1	35.2	6.1**
Third child	48.3	43.5	−4.8	39.0	40.3	1.3
Fourth child and above	72.1	59.4	−12.7*	60.5	57.8	−2.7
All children <1 year	57.7	56.1	−1.6	37.6	43.6	5.9***
**CMR among child 1–5 years**						
First child	14.3	11.8	−2.5	7.1	9.0	1.9
Second child	20.3	9.9	−10.4*	9.1	10.8	1.6
Third child	23.9	19.1	−4.8	15.7	9.9	−5.7
Fourth child and above	38.3	18.6	−19.7***	24.7	17.3	−7.5*
All children 1–5 years	24.0	14.5	−9.6**	11.6	10.9	−0.7
**U5MR among child <5 years**						
First child	72.9	80.4	7.6	42.5	55.1	12.5***
Second child	66.6	55.3	−11.3	37.9	45.6	7.7**
Third child	71.0	61.7	−9.3	54.1	49.8	−4.2
Fourth child and above	107.6	76.8	−30.8***	83.7	74.1	−9.6*
All children <5 years	80.3	69.7	−10.6**	48.8	54.0	5.2**

Abbreviations: CMR, child mortality rates; IMR, infant mortality rates; NMR, neonatal mortality rates; U5MR, under 5 mortality rates.

* *p* < 0.05.

** *p* < 0.01.

*** *p* < 0.001.

**FIGURE 2:**
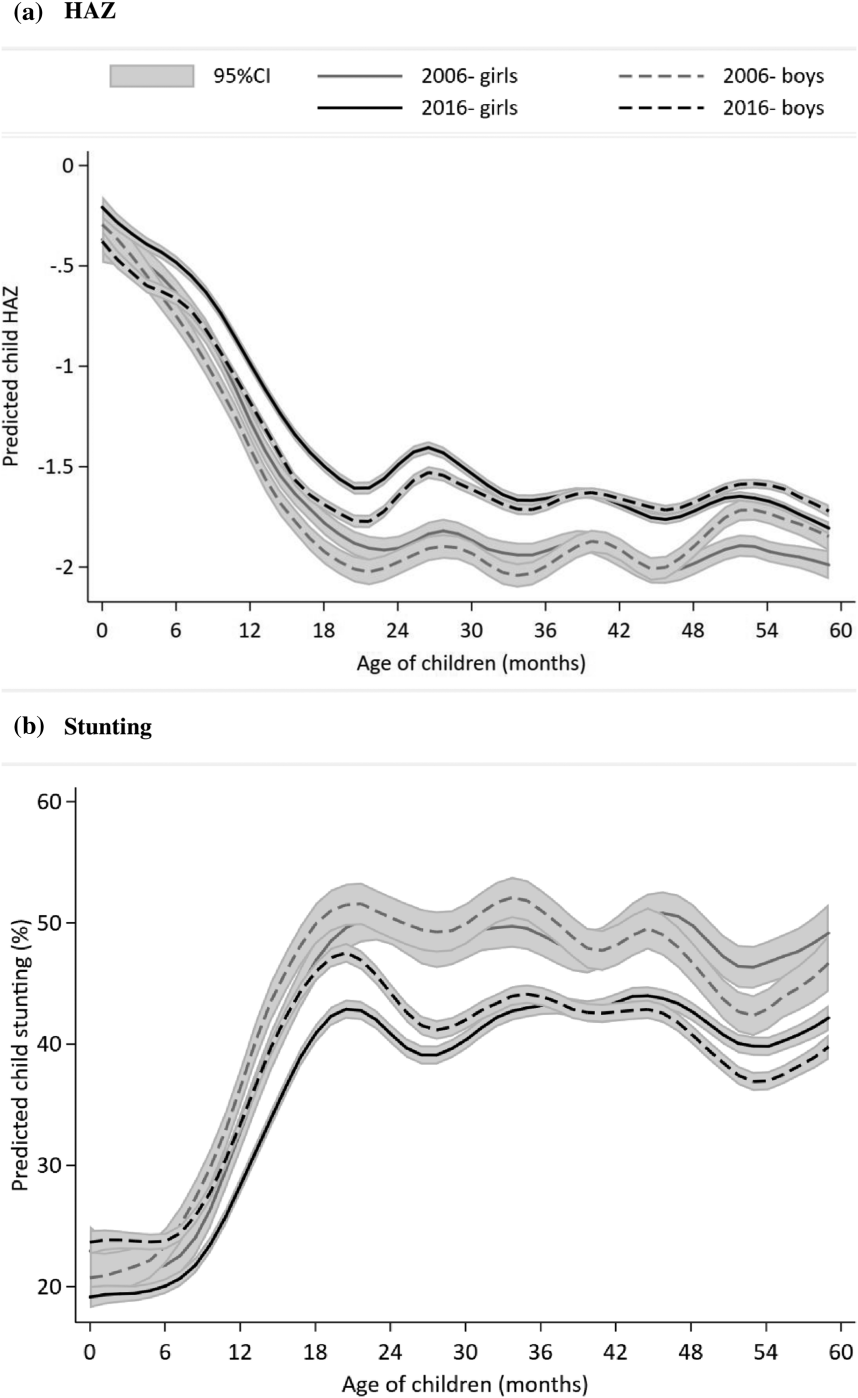
Height‐for‐age *Z*‐scores (HAZ) and stunting, by age and gender in India 2006–2016. (a) HAZ. (b) Stunting. CI, confidence interval

In keeping with common risk factors of mortality and nutrition, patterns in nutrition largely follow those in mortality. As illustrated in Figure 2, average height for age declines with age in months, and stunting rates increase in the manner noted globally (Victora et al., 2010). Younger boys are shorter than girls, but these gaps close as the children approach their second birthdays, after which the curves eventually cross. Finally, the figure confirms that children in the 2016 sample were taller and less likely to be stunted than the corresponding age and gender counterpart in the 2006 sample.

There was a small but significant gender gap in average HAZ for children under 2 in 2016, with boys having lower HAZ than girls (Table 3). The differences in stunting in 2016, however, was appreciable, and, as with average HAZ, the gender gap is slightly to the detriment of boys. In partial contrast, there is no gender difference in HAZ at the national level for children 2–5 years and only a small difference in stunting, with girls at a small disadvantage. Over the entire under 5 population in 2016, girls had higher HAZ and lower stunting than boys (Table 3).

**TABLE 3:** Stunting and HAZ among child <5 years, by gender and state in India 2006–2016

	2006			2016		
	Girls	Boys	Gaps	Girls	Boys	Gaps
**Child <2 years**						
**HAZ**						
First child	−1.25	−1.33	−0.08	−0.87	−1.06	−0.19***
Second child	−1.26	−1.36	−0.11	−0.93	−1.08	−0.15***
Third child	−1.39	−1.59	−0.21*	−1.11	−1.27	−0.15**
Fourth child and above	−1.66	−1.67	−0.01	−1.38	−1.47	−0.09
All children <2 years	−1.37	−1.47	−0.09*	−0.99	−1.16	−0.16***
**Stunting**						
First child	34.5	35.8	1.27	26.9	31.6	4.73***
Second child	35.1	37.3	2.25	29.6	33.6	3.96***
Third child	38.6	43.4	4.83*	33.3	36.6	3.30**
Fourth child and above	46.5	44.6	−1.93	39.8	41.8	2.06
All children <2 years	38.2	39.6	1.5	30.5	34.4	4.0***
**Child 2–5 years**						
**HAZ**						
First child	−1.83	−1.81	0.02	−1.43	−1.46	−0.04
Second child	−2.00	−1.91	0.09	−1.58	−1.60	−0.02
Third child	−2.17	−2.18	0.02	−1.89	−1.74	0.15***
Fourth child and above	−2.46	−2.35	0.10*	−2.12	−2.06	0.06*
All children 2–5 years	−2.10	−2.05	0.05	−1.65	−1.64	0.01
**Stunting**						
First child	44.6	45.1	0.5	35.1	35.6	0.5
Second child	52.4	49.6	−2.8	40.5	39.8	−0.6
Third child	57.8	54.9	−2.9	49.5	44.3	−5.1***
Fourth child and above	63.4	61.0	−2.4	56.0	53.9	−2.1*
All children 2–5 years	53.9	52.4	−1.5	42.0	41.0	−1.0***
**Child 0–5 years**						
**HAZ**						
First child	−1.59	−1.61	−0.03	−1.21	−1.31	−0.10***
Second child	−1.70	−1.70	0.01	−1.32	−1.39	−0.07***
Third child	−1.85	−1.96	−0.11*	−1.59	−1.56	0.03
Fourth child and above	−2.16	−2.11	0.06	−1.85	−1.84	0.01
All children 0–5 years	−1.81	−1.83	−0.02	−1.39	−1.45	−0.06***
**Stunting**						
First child	40.4	41.3	0.9	31.9	34.0	2.1***
Second child	45.5	44.8	−0.7	36.1	37.3	1.2*
Third child	50.0	50.6	0.6	43.1	41.4	−1.7*
Fourth child and above	57.2	55.1	−2.1	50.0	49.4	−0.6
All children 0–5 years	47.6	47.4	−0.2	37.5	38.4	0.9**

Abbreviation: HAZ, height‐for‐age *Z*‐scores.

* *p* < 0.05.

** *p* < 0.01.

*** *p* < 0.001.

Stunting prevalence increases and mean HAZ declines with higher birth order. These patterns are monotonic in all ages and survey years, although the gender difference is not quite monotonic; the point estimate for the gap closes for the relatively few children who are the fourth born or of higher birth order compared with those who are third.

Just as there are differences in overall HAZ and stunting across states, there are differences in gender disparities (Tables S5–S8). The populous states of Bihar and Uttar Pradesh stand out with relatively large gender gaps in stunting, which are statistically significant in the larger 2016 sample. However, in 2016, younger boys were more likely to be stunted in these states, whereas the difference shifts sign for children 2–5. Although few other states have disparities in stunting for children 2–5 years of age, these two large states contribute to a large share of the burden of stunting in India.

## 4 DISCUSSION

Previous literature observed that girls' nutritional status did not improve relative to boys during the periods of economic growth in the 1990s (Tarozzi & Mahajan, 2007), but by the time of the third NFHS, the disparity in nutrition status had largely been eliminated (Corsi et al., 2015). Although a previous study found small improvements in mortality, the probability of mortality for girls was higher than for boys in the north and lower in the south at the end of the last century (Tarozzi & Mahajan, 2007). Our paper, in contrast, indicates that there has been striking progress in child mortality for both boys and girls in India between 2006 and 2016, with slightly more improvement among girls. The improvements in survival among girls eliminated the disparity in mortality after the age of 1. As boys remain slightly more vulnerable in the neonatal period, which accounts for roughly half of all child deaths, this accounts for a higher U5MR for boys relative to girls in 2016.

The current analysis also shows that over the entire under 5 population, girls are not disadvantaged with regard to stature. Indeed, younger boys are more likely to be stunted than are girls. This implies that malnutrition in India is not very different from global patterns. Indeed, using the latest DHS for 68 countries, we found that in 46 of these surveys, boys younger than 5 years had statistically significantly lower HAZ than girls. The other 22 datasets reported no significant differences (19 of these 22 had boys shorter than girls). No sample had standardized heights for girls significantly shorter than boys.

Our results on mortality differentials contrast slightly with a study using UNICEF data, which reported that in 2015, male U5MR exceeded that of females in 193 out of 195 countries (Iqbal et al., 2018), India being an exception. Although more recent UNICEF estimates still show a slightly lower U5MR for boys in India (UNICEF, 2019), the decline in the male to female ratio from 0.94 in 2015 to 0.97 in 2019 is similar to the trend derived from NFHS data indicated in Figure 1 showing a crossover to slightly higher male mortality.

Results for child stature also differ from those in other studies (Jayachandran & Pande, 2017; Oster, 2009). What may account for this difference? One possible reason is that some studies compare *z*‐scores in India with those in Africa despite the fact the *z*‐scores are already standardized relative to the same international norms. As there is a pervasive pattern of boys being shorter than girls in Africa (Svedberg, 1990; Wamani et al., 2007), such a comparison is not based on a neutral benchmark. Another reason for the difference with previous studies is the simple fact that the impressive improvements in stature in India between 2006 and 2016 have been slightly faster for girls. Although the current study does see a small disparity in stunting to the disadvantage of girls of higher birth order relative to boys, this difference is far smaller than the difference in stunting between either boys or girls of earlier birth order and their gender counterparts of latter birth order.

This study is not designed to address why the trends are not parallel, but we can reference reasons that plausibly contribute to the reduction of both gender differences in anthropometry and in mortality. A previous study offers a model postulating that whether gender discrimination is due to market returns to investments, greater propensity of boys to contribute to their parents' household or simply a greater indirect satisfaction of parents from their son's welfare than their daughter's, gender differences in investments should decline as resources increase (Alderman & Gertler, 1997). That study also postulates that households will be more responsive to prices in the case of girls, including changes in the availability of services (Alderman & Gertler, 1997) as occurred in much of India in the decade under consideration. The expansion of health and nutrition services from 2005 onwards reduced the costs of parental investment and thus reduced demand side constraints. Examples of the expansion of services include increased antenatal care during pregnancy and childhood immunization through the National Rural Health Mission. This was accompanied with a large expansion in ICDS, a nutrition programme that is used predominantly by poorer households (Chakrabarti et al., 2019). Although we do not see gender differentials in access to these services, the impacts of such large service expansions are likely society wide. In addition to the expansion of health and nutrition programmes, many states also rolled out a range of cash incentive programmes targeted towards the education and support of girls (Sekher, 2012).

Researchers have explored the origins and persistence of son preferences in terms of cultural features including religion and patrilocal inheritance patterns across regions of India (Jain, 2014). Although the current study does not attempt to parse out the relative contribution of culture, we note that culture seldom changes as rapidly as did relative mortality rates in the period studied. In contrast, improved access to health services has been dynamic. Although a portion of gender difference in anthropometry reflects anticipation of future child investments, the main contribution to nutritional differences accrues in children of higher birth order (Jayachandran & Pande, 2017). This is not necessarily direct discrimination of later born children but rather a reflection that birth order and the number of siblings are correlated with household resources (Spears et al., 2019). This, again, implies that economic progress—as well as any other trends that result in reduced fertility—will also reduce gender imbalances.

How much of the reduction in gender disparity is due to trends in fertility? We explored this by assuming that the share of children born in different birth orders in 2006 was the same as observed in 2016. That is, we assume that a larger share of children was first or second born than actually was observed in the earlier year. We also assumed that the mortality rates and stunting prevalence per gender and birth order remained as they were in 2006. Shifting to this scenario, however, makes very little difference. We estimated only a drop in overall mortality from 74.1 to 72.5 and stunting from 47.2% to 46.2%. This is about 6% of the total decline in mortality and 2% of the total decline in stunting. Although this estimate does not take into account that birth order is related to overall family size, which is not included in this exercise, it illustrates that the direct contribution of birth order changes over the decade to the favourable mortality and nutrition trends is modest.

This ties to an additional possible reason that improvements in mortality and nutritional status have been faster for girls than boys in recent years (Anukriti et al., 2020), and the availability of sex selection technology has benefited survival rates of girls by 2006 additional to any improvements in health services that benefit both genders. To the degree that this occurs, it contributes to the observed decline. But because mortality rates are defined as a share of live births, the reported mortality that is analysed here is not a biased estimate even if it differs from a counterfactual of what might have occurred in the absence of ultrasound technology (Anukriti et al., 2020; Hu & Schlosser, 2015). These authors do not deny the prevalence of son preference but claim that the preference is manifested differently with modern technology. Nor do the authors downplay other negative consequences of sex selection (Amaral & Bhalotra, 2017). Similarly, we do not view these improvements in the survival and nutrition of girls as the final step in reducing disparities. Many remain; the government needs to continue to address these, both in the supply of services and in changing incentives for education and later marriage (Sekher, 2012).

The question of the degree that sex‐selective abortions drive the trends in mortality and nutrition reported here hinges, in part, on the question of whether there has been a change in the prevalence of such practices between 2006 and 2016. Using a previous criterion reported in India (Jha et al., 2011), the sex ratio of the second‐born child conditional on the gender of the first, there is no apparent trend in the selective abortions. In the 2006 survey, there were 99 boys born for every 100 girls following the birth of a male first child and 118 if the first child was female. In 2016, these ratios were 108 and 110, respectively. Although these numbers raise questions well outside the current study, they provide support to the view that the changes in gender disparities in mortality and nutrition between survey rounds are not primarily a reflection of access to prenatal determination of the child's gender.

A limitation of this study stems from the fact that anthropometry can only be collected on survivors. If there are gender‐specific trends in mortality between periods studied, this will have an effect on trends in anthropometry (Alderman et al., 2011; Harttgen et al., 2019). It is unlikely, however, that the different rates of improvements in survival appreciably mask or obscure the patterns in anthropometry discussed here. In particular, improved survival has a tendency to slightly increase stunting in the overall population; thus, the greater improvements in CMR and U5MR among girls would somewhat reduce any bias relative to the earlier period. Because the bias on HAZ from not observing heights of children who died prior to the survey is upwards, the decrease in mortality implies a small downward impact on observed changes in stature. In the absence of any bias in both periods, the measured improvements in HAZ for girls would have been slightly larger relative to boys. Similar expectations can be made regarding any small bias in stunting, which in this case reduces observed stunting due to loss of stunted children from the sample. Likewise, the more rapid decline in IMR for girls attenuates rather than exaggerates the relative improvement in measured stunting for girls under 2 over the period studied. Mortality may also be slightly underestimated because many children have not lived through the risk period covered in the mortality statistic. That is, if a child is only 36 months at the time of the survey, we cannot tell if s/he will survive to 60 months. However, this should not have a strong effect on a comparison of relative disparity over time (Karlsson et al., 2019).

## 5 CONCLUSIONS

India remains a challenging country to be a girl. At the same time, our research demonstrates that the basic biological disadvantages of sheer survival or of being undernourished can change and quite dramatically. The reasons behind the reduced sex differentials need more exploration, but our analyses are a step in identifying states where further investigation would be useful. It is more likely than not that a complex set of positive forces—improvements in health services, improvements in household conditions and changes in societal perceptions—came together to improve survival chances and physical growth for millions of girls in India. Staying the course to ensure that economic, social and health policies remain in place to support further improvements in health and nutrition will be important for India's girls; this is especially important in the context of massive disruptions like the COVID‐19 pandemic. Similar efforts to understand and close disparities in education can build upon such progress.

## CONFLICTS OF INTEREST

The authors declare no conflicts of interest.

## CONTRIBUTIONS

HA conceived the idea for the manuscript, supported data interpretation and wrote significant sections of the manuscript. PHN conceived the idea for the manuscript, conducted the statistical analysis and wrote significant sections of the manuscript. LMT conducted the statistical analysis and reviewed and edited the manuscript. PM supported data interpretation and reviewed and edited the manuscript. All authors read and approved the final submitted manuscript.

## Supporting information


**Figure S1** Child mortality rate by gender and survey year in India, 2006–2016
**Table S1**: Neonatal mortality rate among children < 1 month, by gender and state in India 2006–2016
**Table S2**: Infant mortality rate among children <1 year, by gender and state in India 2006–2016
**Table S3**: Child mortality rate among children 1–5 years, by gender and state in India 2006–2016
**Table S4**: Under 5 mortality rates among children <5 years, by gender and state in India 2006–2016
**Table S5**: HAZ among child <2 years, by gender and state in India 2006–2016
**Table S6**: Stunting among child <2 years, by gender and states in India 2006–2016
**Table S7**: HAZ among child 2–5 years, by gender and state in India 2006–2016
**Table S8**: Stunting among child 2–5 years, by gender and state in India 2006–2016

## Data Availability

The data that support the findings of this study are available in the tables/figures and supporting information of this article. Additional data are available upon request.
