# Supplementary material for: Trends and geographic variability in gender inequalities in child mortality and stunting in India, 2006–2016
Source: Matern Child Nutr. 2021 Mar 14;17(3):e13179. doi: 10.1111/mcn.13179 (PMC8189201; doi:10.1111/mcn.13179)
Supplement: Supplementary file 1 — Figure S1 Child mortality rate by gender and survey year in India, 2006–2016 Table S1: Neonatal mortality rate among children < 1 month, by gender and state in India 2006–2016 Table S2: Infant mortality rate among children <1 year, by gender and state in India 2006–2016 Table S3: Child mortality rate among children 1–5 years, by gender and state in India 2006–2016 Table S4: Under 5 mortality rates among children <5 years, by gender and state in India 2006–2016 Table S5: HAZ among child <2 years, by gender and state in India 2006–2016 Table S6: Stunting among child <2 years, by gender and states in India 2006–2016 Table S7: HAZ among child 2–5 years, by gender and state in India 2006–2016 Table S8: Stunting among child 2–5 years, by gender and state in India 2006–2016 [file MCN-17-e13179-s001.docx]

**Supplementary Figure 1: Child mortality rate by gender and survey year in India, 2006-2016**

| 1. **NMR** | 1. **IMR** |
| --- | --- |
|  |  |
| 1. **CMR** | 1. **U5 MR** |
|  |  |

**Supplementary Table 1: Neonatal mortality rate among children < 1 month, by gender and state in India 2006-2016**

|  | **2006** | | | **2016** | | |
| --- | --- | --- | --- | --- | --- | --- |
|  | **Girls** | **Boys** | **Gaps** | **Girls** | **Boys** | **Gaps** |
| A&N Islands |  |  |  | 9.9 | 2.9 | -6.9 |
| Andhra Pradesh | 40.9 | 40.9 | 0 | 16.4 | 25.5 | 9.1* |
| Arunachal Pradesh | 22.2 | 47.9 | 25.7* | 14.2 | 9.4 | -4.8 |
| Assam | 45.9 | 45.6 | -0.3 | 28.8 | 36.7 | 7.9 |
| Bihar | 36.7 | 42.9 | 6.2 | 31.3 | 41.8 | 10.5*** |
| Chandigarh |  |  |  | 40.7 | 20.4 | -20.3 |
| Chhattisgarh | 40.9 | 62.1 | 21.1 | 37.5 | 46.6 | 9.1* |
| D&N Haveli |  |  |  | 5.5 | 13 | 7.4 |
| Daman & Diu |  |  |  | 20.8 | 29.5 | 8.7 |
| Delhi | 30.3 | 30.2 | -0.1 | 20.1 | 13.4 | -6.7 |
| Goa | 12.2 | 6.2 | -6 | 3.5 | 22.5 | 19 |
| Gujarat | 36.5 | 31.1 | -5.4 | 21.4 | 31.6 | 10.1* |
| Haryana | 23.1 | 24.5 | 1.4 | 20.6 | 22.9 | 2.3 |
| Himachal Pradesh | 32 | 24.7 | -7.3 | 18.5 | 29.4 | 10.9 |
| Jammu & Kashmir | 29 | 31.8 | 2.8 | 21.5 | 24.7 | 3.2 |
| Jharkhand | 43.4 | 52.6 | 9.2 | 29.2 | 36.4 | 7.1* |
| Karnataka | 25.1 | 31 | 5.9 | 16.2 | 21 | 4.8 |
| Kerala | 12.3 | 11.4 | -0.9 | 5.3 | 3.8 | -1.5 |
| Lakshadweep |  |  |  | 35.3 | 12.9 | -22.4 |
| Madhya Pradesh | 48 | 42.4 | -5.5 | 31.8 | 41.2 | 9.4*** |
| Maharashtra | 24.6 | 34.6 | 10 | 12.1 | 19.9 | 7.8* |
| Manipur | 11.2 | 26.2 | 14.9* | 13.3 | 17.9 | 4.5 |
| Meghalaya | 18.6 | 27.9 | 9.3 | 14.7 | 22.5 | 7.8 |
| Mizoram | 16 | 14.6 | -1.4 | 13.2 | 9.6 | -3.6 |
| Nagaland | 20.8 | 18.3 | -2.5 | 14.9 | 18.1 | 3.2 |
| Orissa | 33.8 | 56.1 | 22.2* | 29 | 27.7 | -1.3 |
| Pondicherry |  |  |  | 3 | 8.5 | 5.4 |
| Punjab | 25.7 | 29.5 | 3.8 | 23.4 | 20.1 | -3.2 |
| Rajasthan | 48.9 | 39 | -9.9 | 28.3 | 30.9 | 2.7 |
| Sikkim | 24.1 | 15.9 | -8.2 | 11.2 | 27.2 | 16 |
| Tamil Nadu | 19.6 | 18.9 | -0.7 | 13.4 | 15 | 1.7 |
| Tripura | 24.9 | 40.4 | 15.4 | 10.9 | 15.5 | 4.6 |
| Uttar Pradesh | 45.5 | 49.6 | 4.1 | 40.8 | 49.3 | 8.5** |
| Uttaranchal | 21.4 | 33.5 | 12 | 25.4 | 28.7 | 3.3 |
| West Bengal | 28 | 47.6 | 19.6* | 14.7 | 29.7 | 15.0** |
| **India** | **36.7** | **41.0** | **4.3** | **25.7** | **32.9** | **7.3***** |

*p<0.05, **p<0.01, ***p<0.001

**Supplementary Table 2: Infant mortality rate among children <1 year, by gender and state in India 2006-2016**

|  | **2006** | | | **2016** | | |
| --- | --- | --- | --- | --- | --- | --- |
|  | **Girls** | **Boys** | **Gaps** | **Girls** | **Boys** | **Gaps** |
| A&N Islands |  |  |  | 15.3 | 3.2 | -12.1 |
| Andhra Pradesh | 52.8 | 54.4 | 1.7 | 26.4 | 34.5 | 8.1 |
| Arunachal Pradesh | 44.8 | 78.9 | 34.1* | 23.4 | 22.4 | -1.1 |
| Assam | 63 | 70.5 | 7.5 | 43.2 | 52 | 8.8 |
| Bihar | 68.1 | 57.8 | -10.3 | 43.2 | 53 | 9.8*** |
| Chandigarh |  |  |  | 40.7 | 31.7 | -9 |
| Chhattisgarh | 61.4 | 82 | 20.6 | 50.6 | 57.2 | 6.5 |
| D&N Haveli |  |  |  | 19.5 | 48.5 | 29 |
| Daman & Diu |  |  |  | 29.2 | 38.4 | 9.3 |
| Delhi | 46.7 | 36.4 | -10.4 | 39 | 22.1 | -16.8 |
| Goa | 21.9 | 8.5 | -13.3 | 3.5 | 22.5 | 19 |
| Gujarat | 54.1 | 47.3 | -6.8 | 29.3 | 37.7 | 8.4 |
| Haryana | 42.2 | 42.6 | 0.3 | 34 | 31.3 | -2.7 |
| Himachal Pradesh | 35 | 36 | 1 | 27.4 | 39.4 | 12 |
| Jammu & Kashmir | 45.8 | 45.3 | -0.5 | 28.6 | 36.2 | 7.6 |
| Jharkhand | 64.4 | 73.9 | 9.5 | 41.8 | 46.1 | 4.3 |
| Karnataka | 38 | 46.4 | 8.4 | 23.9 | 30.7 | 6.8 |
| Kerala | 14.5 | 15.7 | 1.1 | 5.7 | 5.5 | -0.2 |
| Lakshadweep |  |  |  | 35.3 | 20.4 | -14.9 |
| Madhya Pradesh | 72.8 | 62.1 | -10.6 | 46.1 | 55.2 | 9.1** |
| Maharashtra | 33.4 | 38 | 4.6 | 19.2 | 27.6 | 8.4* |
| Manipur | 28.4 | 31.3 | 2.9 | 18.9 | 24 | 5.2 |
| Meghalaya | 35.3 | 54.6 | 19.3 | 25.7 | 34.6 | 8.8 |
| Mizoram | 40.8 | 25.1 | -15.7 | 42.4 | 34.6 | -7.8 |
| Nagaland | 36 | 40.6 | 4.6 | 29.7 | 30.3 | 0.6 |
| Orissa | 55.1 | 70 | 15 | 38.7 | 40.2 | 1.5 |
| Pondicherry |  |  |  | 13.8 | 20.7 | 6.9 |
| Punjab | 45.4 | 39.8 | -5.6 | 34.6 | 26.4 | -8.2 |
| Rajasthan | 76.8 | 56.2 | -20.7 | 39.6 | 42.6 | 3 |
| Sikkim | 38.6 | 29.6 | -8.9 | 23.8 | 33.3 | 9.5 |
| Tamil Nadu | 36.3 | 27.4 | -8.9 | 18.3 | 21.6 | 3.2 |
| Tripura | 31.6 | 70.8 | 39.2* | 20.5 | 33.8 | 13.2 |
| Uttar Pradesh | 75 | 70.6 | -4.4 | 62.6 | 64.8 | 2.3 |
| Uttaranchal | 33.1 | 47.9 | 14.8 | 35.7 | 42.4 | 6.8 |
| West Bengal | 40.4 | 54.6 | 14.2 | 21.1 | 35.4 | 14.3** |
| **India** | **57.7** | **56.1** | **-1.6** | **37.6** | **43.6** | **5.9***** |

*p<0.05, **p<0.01, ***p<0.001

**Supplementary Table 3: Child mortality rate among children 1-5 years, by gender and state in India 2006-2016**

|  | **2006** | | | **2016** | | |
| --- | --- | --- | --- | --- | --- | --- |
|  | **Girls** | **Boys** | **Gaps** | **Girls** | **Boys** | **Gaps** |
| A&N Islands |  |  |  | 0.0 | 0.0 | 0.0 |
| Andhra Pradesh | 6.1 | 22.2 | 16.1 | 3.8 | 4.3 | 0.4 |
| Arunachal Pradesh | 33.2 | 20.6 | -12.6 | 8.7 | 19.2 | 10.5 |
| Assam | 10.7 | 45.9 | 35.1 | 9.6 | 8.5 | -1.2 |
| Bihar | 13.7 | 16.9 | 3.1 | 12.7 | 9.1 | -3.6 |
| Chandigarh |  |  |  | 0 | 0 | 0 |
| Chhattisgarh | 26.9 | 18.9 | -8 | 14.3 | 9.9 | -4.4 |
| D&N Haveli |  |  |  | 8.2 | 0 | -8.2 |
| Daman & Diu |  |  |  | 0 | 0 | 0 |
| Delhi | 4.8 | 11.4 | 6.6 | 6.1 | 16.8 | 10.8 |
| Goa | 3.5 | 0 | -3.5 | 0 | 0 | 0 |
| Gujarat | 25.8 | 9.2 | -16.6 | 13.7 | 8.7 | -5 |
| Haryana | 17 | 7.6 | -9.3 | 12.7 | 4.6 | -8.1 |
| Himachal Pradesh | 3.9 | 8.4 | 4.5 | 3.4 | 9 | 5.5 |
| Jammu & Kashmir | 16.3 | 2 | -14.3 | 13.5 | 18.9 | 5.3 |
| Jharkhand | 30.5 | 8.7 | -21.8 | 14.8 | 10.7 | -4.1 |
| Karnataka | 12.5 | 4.5 | -8 | 8.7 | 10.6 | 2 |
| Kerala | 4.3 | 0 | -4.3 | 2.1 | 7.1 | 5 |
| Lakshadweep |  |  |  | 0 | 0 | 0 |
| Madhya Pradesh | 25.5 | 30.2 | 4.7 | 16.6 | 11 | -5.6* |
| Maharashtra | 11.6 | 8.7 | -2.9 | 4.7 | 6.4 | 1.7 |
| Manipur | 17 | 12.4 | -4.6 | 2.2 | 3.6 | 1.4 |
| Meghalaya | 13.8 | 18.9 | 5.1 | 15.9 | 13.8 | -2.1 |
| Mizoram | 40.9 | 7.2 | -33.7 | 12.1 | 4.5 | -7.7 |
| Nagaland | 24.6 | 32.8 | 8.2 | 11.4 | 19.9 | 8.5 |
| Orissa | 14.5 | 18.3 | 3.8 | 5.9 | 12.3 | 6.3 |
| Pondicherry |  |  |  | 0.7 | 2 | 1.3 |
| Punjab | 14.5 | 6.7 | -7.8 | 0.2 | 4 | 3.8* |
| Rajasthan | 35.7 | 12.9 | -22.8 | 11.2 | 10.3 | -0.9 |
| Sikkim | 6 | 0 | -6 | 6 | 0 | -6 |
| Tamil Nadu | 13.7 | 4.5 | -9.1 | 17.7 | 32.2 | 14.5 |
| Tripura | 5.2 | 5.3 | 0 | 12.3 | 2.2 | -10.1 |
| Uttar Pradesh | 47 | 13.7 | -33.3*** | 19.4 | 13.9 | -5.6 |
| Uttaranchal | 13.9 | 59.1 | 45.2 | 14.1 | 5.7 | -8.4 |
| West Bengal | 22.6 | 10.1 | -12.4 | 3.6 | 7.6 | 4 |
| **India** | **24.0** | **14.5** | **-9.6**** | **11.6** | **10.9** | **-0.7** |

*p<0.05, **p<0.01, ***p<0.001

**Supplementary Table 4: Under 5 mortality rates among children <5 years, by gender and state in India 2006-2016**

|  | **2006** | | | **2016** | | |
| --- | --- | --- | --- | --- | --- | --- |
|  | **Girls** | **Boys** | **Gaps** | **Girls** | **Boys** | **Gaps** |
| A&N Islands |  |  |  | 15.3 | 3.2 | -12.1 |
| Andhra Pradesh | 58.6 | 75.4 | 16.9 | 30.2 | 38.6 | 8.4 |
| Arunachal Pradesh | 76.5 | 97.8 | 21.3 | 31.9 | 41.2 | 9.2 |
| Assam | 73 | 113.1 | 40 | 52.4 | 60.1 | 7.6 |
| Bihar | 80.9 | 73.7 | -7.2 | 55.4 | 61.6 | 6.2 |
| Chandigarh |  |  |  | 40.7 | 31.7 | -9 |
| Chhattisgarh | 86.7 | 99.4 | 12.7 | 64.2 | 66.5 | 2.3 |
| D&N Haveli |  |  |  | 27.6 | 48.5 | 20.9 |
| Daman & Diu |  |  |  | 29.2 | 38.4 | 9.3 |
| Delhi | 51.3 | 47.4 | -4 | 44.8 | 38.6 | -6.2 |
| Goa | 25.3 | 8.5 | -16.8 | 3.5 | 22.5 | 19 |
| Gujarat | 78.6 | 56 | -22.5 | 42.6 | 46.1 | 3.4 |
| Haryana | 58.5 | 49.9 | -8.6 | 46.2 | 35.8 | -10.4 |
| Himachal Pradesh | 38.8 | 44.1 | 5.4 | 30.8 | 48 | 17.2 |
| Jammu & Kashmir | 61.3 | 47.2 | -14.2 | 41.7 | 54.4 | 12.7 |
| Jharkhand | 93 | 82 | -11 | 56 | 56.3 | 0.3 |
| Karnataka | 50 | 50.7 | 0.6 | 32.3 | 41 | 8.6 |
| Kerala | 18.7 | 15.7 | -3.1 | 7.7 | 12.5 | 4.8 |
| Lakshadweep |  |  |  | 35.3 | 20.4 | -14.9 |
| Madhya Pradesh | 96.4 | 90.4 | -6 | 62 | 65.6 | 3.6 |
| Maharashtra | 44.6 | 46.4 | 1.7 | 23.8 | 33.9 | 10.1* |
| Manipur | 44.9 | 43.3 | -1.6 | 21 | 27.6 | 6.6 |
| Meghalaya | 48.5 | 72.4 | 23.9 | 41.2 | 47.9 | 6.7 |
| Mizoram | 80 | 32.1 | -47.9 | 54 | 38.9 | -15.1 |
| Nagaland | 59.7 | 72 | 12.3 | 40.8 | 49.7 | 8.9 |
| Orissa | 68.7 | 87 | 18.3 | 44.4 | 51.9 | 7.5 |
| Pondicherry |  |  |  | 14.5 | 22.6 | 8.1 |
| Punjab | 59.2 | 46.2 | -13 | 34.8 | 30.3 | -4.5 |
| Rajasthan | 109.8 | 68.4 | -41.4** | 50.4 | 52.5 | 2.1 |
| Sikkim | 44.3 | 29.6 | -14.7 | 29.7 | 33.3 | 3.6 |
| Tamil Nadu | 49.4 | 31.8 | -17.6 | 35.7 | 53.1 | 17.4 |
| Tripura | 36.7 | 75.7 | 39.0* | 32.5 | 35.9 | 3.4 |
| Uttar Pradesh | 118.5 | 83.4 | -35.1** | 80.8 | 77.8 | -3 |
| Uttaranchal | 46.6 | 104.2 | 57.6 | 49.3 | 47.9 | -1.4 |
| West Bengal | 62.1 | 64.2 | 2.1 | 24.6 | 42.7 | 18.1** |
| **India** | **80.3** | **69.7** | **-10.6**** | **48.8** | **54.0** | **5.2**** |

*p<0.05, **p<0.01, ***p<0.001

**Supplementary Table 5: HAZ among child <2 years, by gender and state in India 2006-2016**

|  | **2006** | | | **2016** | | |
| --- | --- | --- | --- | --- | --- | --- |
|  | **Girls** | **Boys** | **Gaps** | **Girls** | **Boys** | **Gaps** |
| A&N Islands |  |  |  | -0.6 | -0.9 | -0.3 |
| Andhra Pradesh | -1.3 | -1.3 | 0 | -0.7 | -0.8 | -0.1 |
| Arunachal Pradesh | -0.7 | -1.1 | -0.4 | -0.6 | -0.9 | -0.3* |
| Assam | -1.2 | -1.5 | -0.3 | -1 | -1.2 | -0.2** |
| Bihar | -1.6 | -1.6 | 0 | -1.2 | -1.4 | -0.1** |
| Chandigarh |  |  |  | -0.6 | -0.8 | -0.2 |
| Chhattisgarh | -1.7 | -1.8 | 0 | -1.1 | -1.4 | -0.3*** |
| D&N Haveli |  |  |  | -1.7 | -1.3 | 0.4 |
| Daman & Diu |  |  |  | 0.2 | 0.3 | 0.1 |
| Delhi | -1.2 | -1.3 | -0.1 | -0.8 | -0.9 | -0.1 |
| Goa | -0.8 | -1.1 | -0.4 | 0 | -0.7 | -0.7 |
| Gujarat | -1.5 | -1.7 | -0.2 | -0.7 | -1 | -0.3** |
| Haryana | -1.3 | -1.8 | -0.4** | -0.8 | -1 | -0.2 |
| Himachal Pradesh | -0.8 | -1.1 | -0.3 | -0.6 | -0.8 | -0.3* |
| Jammu & Kashmir | -1.1 | -0.9 | 0.2 | -0.4 | -0.6 | -0.2* |
| Jharkhand | -1.4 | -1.7 | -0.3 | -1.1 | -1.3 | -0.2*** |
| Karnataka | -1.3 | -1.3 | 0 | -1 | -1.2 | -0.3* |
| Kerala | -0.7 | -1.1 | -0.3 | -0.3 | -0.8 | -0.5** |
| Lakshadweep |  |  |  | -1.3 | -1.2 | 0.1 |
| Madhya Pradesh | -1.3 | -1.7 | -0.3* | -1 | -1.2 | -0.2*** |
| Maharashtra | -1.3 | -1.5 | -0.2 | -0.7 | -0.8 | -0.1 |
| Manipur | -0.6 | -0.8 | -0.2 | -0.8 | -1 | -0.2* |
| Meghalaya | -0.7 | -1.1 | -0.5 | -0.8 | -1.1 | -0.3* |
| Mizoram | -0.7 | -1.3 | -0.5* | -0.6 | -0.7 | -0.1 |
| Nagaland | -0.8 | -0.9 | 0 | -0.4 | -0.6 | -0.2 |
| Orissa | -1.6 | -1.4 | 0.1 | -1 | -1.1 | -0.1 |
| Pondicherry |  |  |  | -0.1 | -0.2 | -0.1 |
| Punjab | -1.2 | -1.1 | 0.1 | -0.7 | -0.9 | -0.2 |
| Rajasthan | -1.2 | -1.2 | 0 | -1 | -1.2 | -0.2** |
| Sikkim | -0.7 | -0.9 | -0.2 | -0.3 | -0.6 | -0.3 |
| Tamil Nadu | -0.5 | -0.9 | -0.3 | -0.7 | -0.9 | -0.2 |
| Tripura | -1.1 | -1.3 | -0.2 | -0.5 | -0.2 | 0.3 |
| Uttar Pradesh | -1.6 | -1.6 | 0 | -1.3 | -1.4 | -0.1*** |
| Uttaranchal | -1.5 | -1.4 | 0.1 | -0.9 | -0.9 | -0.1 |
| West Bengal | -1.3 | -1.3 | 0 | -1.1 | -1.1 | 0 |
| **India** | **-1.4** | **-1.5** | **-0.1*** | **-1** | **-1.2** | **-0.2***** |

*p<0.05, **p<0.01, ***p<0.001

**Supplementary Table 6: Stunting among child <2 years, by gender and states in India 2006-2016**

|  | **2006** | | | **2016** | | |
| --- | --- | --- | --- | --- | --- | --- |
|  | **Girls** | **Boys** | **Gaps** | **Girls** | **Boys** | **Gaps** |
| A&N Islands |  |  |  | 20.9 | 23.9 | 2.9 |
| Andhra Pradesh | 31.5 | 34 | 2.5 | 21.2 | 22.5 | 1.3 |
| Arunachal Pradesh | 25.2 | 29.6 | 4.4 | 26.4 | 31 | 4.7 |
| Assam | 33.2 | 38 | 4.8 | 28.9 | 35.3 | 6.4*** |
| Bihar | 44.2 | 40.6 | -3.6 | 36.9 | 39.6 | 2.7* |
| Chandigarh |  |  |  | 14.9 | 29.2 | 14.3 |
| Chhattisgarh | 45.6 | 48.1 | 2.5 | 31 | 37.7 | 6.7** |
| D&N Haveli |  |  |  | 47.1 | 44.9 | -2.2 |
| Daman & Diu |  |  |  | 14.3 | 13.3 | -1 |
| Delhi | 37.7 | 37.8 | 0.1 | 24.1 | 28.7 | 4.6 |
| Goa | 20.6 | 24.7 | 4.1 | 15.3 | 25.8 | 10.5 |
| Gujarat | 42.1 | 43.3 | 1.2 | 27.9 | 33.6 | 5.7** |
| Haryana | 37.2 | 44.2 | 7 | 26 | 34 | 8.0*** |
| Himachal Pradesh | 25.1 | 31.9 | 6.8 | 16.2 | 24.2 | 8.1** |
| Jammu & Kashmir | 34.6 | 24.2 | -10.5* | 21.8 | 26.9 | 5.1* |
| Jharkhand | 39.6 | 44.3 | 4.7 | 33.6 | 40.2 | 6.7*** |
| Karnataka | 33.7 | 37.6 | 3.9 | 33.1 | 39.6 | 6.5* |
| Kerala | 24.3 | 29.2 | 4.9 | 18.7 | 23.1 | 4.4 |
| Lakshadweep |  |  | 0 | 24.2 | 35 | 10.8 |
| Madhya Pradesh | 38.7 | 43.4 | 4.8 | 32 | 35.6 | 3.6** |
| Maharashtra | 38.4 | 40.1 | 1.7 | 27.8 | 31 | 3.3 |
| Manipur | 20.6 | 25.7 | 5.1 | 21.1 | 25.9 | 4.8* |
| Meghalaya | 33.6 | 35.3 | 1.7 | 28.4 | 34 | 5.6* |
| Mizoram | 25.9 | 31.4 | 5.5 | 18.6 | 18.2 | -0.4 |
| Nagaland | 27.2 | 30.1 | 2.9 | 16.1 | 22 | 5.9* |
| Orissa | 39.6 | 39.7 | 0.1 | 28.8 | 32.7 | 3.9* |
| Pondicherry |  |  |  | 17.2 | 18 | 0.8 |
| Punjab | 34.9 | 34.9 | 0 | 21.7 | 25.6 | 3.9 |
| Rajasthan | 32.3 | 35.2 | 2.9 | 30.9 | 35.1 | 4.2** |
| Sikkim | 27.3 | 33.1 | 5.9 | 22.2 | 27.9 | 5.7 |
| Tamil Nadu | 27.4 | 30.6 | 3.1 | 26.7 | 33.1 | 6.5** |
| Tripura | 25.7 | 32.7 | 6.9 | 19.2 | 17.4 | -1.8 |
| Uttar Pradesh | 44 | 44.2 | 0.1 | 34.7 | 38.5 | 3.7** |
| Uttaranchal | 36.6 | 37.7 | 1.1 | 30.8 | 31.9 | 1.1 |
| West Bengal | 33.7 | 36.5 | 2.7 | 30 | 30.7 | 0.7 |
| **India** | **38.2** | **39.6** | **1.5** | **30.5** | **34.4** | **4.0***** |

*p<0.05, **p<0.01, ***p<0.001

**Supplementary Table 7: HAZ among child 2-5 years, by gender and state in India 2006-2016**

|  | **2006** | | | **2016** | | |
| --- | --- | --- | --- | --- | --- | --- |
|  | **Girls** | **Boys** | **Gaps** | **Girls** | **Boys** | **Gaps** |
| A&N Islands |  |  |  | -1.2 | -0.9 | 0.3 |
| Andhra Pradesh | -1.9 | -1.8 | 0.1 | -1.5 | -1.6 | 0 |
| Arunachal Pradesh | -1.8 | -2 | -0.2 | -1 | -1.2 | -0.2* |
| Assam | -2.1 | -1.9 | 0.1 | -1.5 | -1.6 | -0.1 |
| Bihar | -2.6 | -2.4 | 0.2* | -2.1 | -2 | 0.1 |
| Chandigarh |  |  |  | -1.4 | -1.6 | -0.2 |
| Chhattisgarh | -2.2 | -2.2 | 0 | -1.7 | -1.7 | -0.1 |
| D&N Haveli |  |  |  | -1.2 | -1.5 | -0.3 |
| Daman & Diu |  |  |  | -0.8 | -0.8 | -0.1 |
| Delhi | -1.6 | -1.8 | -0.2 | -1.3 | -1.4 | -0.1 |
| Goa | -1.3 | -1 | 0.3 | -1.3 | -0.7 | 0.6* |
| Gujarat | -2.2 | -2.2 | 0 | -1.7 | -1.6 | 0.1* |
| Haryana | -2 | -1.8 | 0.2 | -1.3 | -1.5 | -0.1 |
| Himachal Pradesh | -1.8 | -1.9 | -0.2 | -1.4 | -1.4 | 0 |
| Jammu & Kashmir | -1.6 | -1.4 | 0.2 | -1.3 | -1.1 | 0.1 |
| Jharkhand | -2.1 | -2.1 | 0 | -1.8 | -1.8 | 0 |
| Karnataka | -1.8 | -1.8 | 0 | -1.3 | -1.3 | 0 |
| Kerala | -1.1 | -1.3 | -0.1 | -0.7 | -0.7 | 0 |
| Lakshadweep |  |  |  | -1.6 | -1.2 | 0.4* |
| Madhya Pradesh | -2.2 | -2.2 | 0 | -1.8 | -1.8 | 0 |
| Maharashtra | -1.9 | -2 | 0 | -1.4 | -1.4 | 0 |
| Manipur | -1.8 | -1.8 | 0.1 | -1.5 | -1.4 | 0.1 |
| Meghalaya | -2.1 | -2.2 | -0.1 | -1.7 | -1.9 | -0.2** |
| Mizoram | -1.7 | -2 | -0.2 | -1.5 | -1.5 | 0 |
| Nagaland | -1.7 | -1.8 | -0.1 | -1.3 | -1.5 | -0.2** |
| Orissa | -2 | -1.8 | 0.2** | -1.5 | -1.5 | 0.1 |
| Pondicherry |  |  |  | -0.9 | -1.2 | -0.3 |
| Punjab | -1.7 | -1.7 | 0 | -1.1 | -1.2 | -0.1 |
| Rajasthan | -2 | -2 | 0 | -1.6 | -1.7 | -0.1* |
| Sikkim | -1.6 | -1.8 | -0.2 | -1.1 | -1.3 | -0.2 |
| Tamil Nadu | -1.1 | -1.3 | -0.2 | -1 | -1 | 0 |
| Tripura | -1.7 | -1.5 | 0.2 | -1.2 | -1.3 | -0.1 |
| Uttar Pradesh | -2.5 | -2.5 | 0.1 | -2 | -2 | 0.1* |
| Uttaranchal | -2 | -2.1 | -0.2 | -1.3 | -1.3 | 0 |
| West Bengal | -1.9 | -1.9 | 0 | -1.6 | -1.5 | 0.1 |
| **India** | **-2.1** | **-2.05** | **0.05** | **-1.6** | **-1.6** | **0.01** |

*p<0.05, **p<0.01, ***p<0.001

**Supplementary Table 8: Stunting among child 2-5 years, by gender and state in India 2006-2016**

|  | **2006** | | | **2016** | | |  |
| --- | --- | --- | --- | --- | --- | --- | --- |
|  | **Girls** | **Boys** | **Gaps** | **Girls** | **Boys** | **Gaps** | |
| A&N Islands |  |  |  | 25.7 | 21.1 | -4.6 | |
| Andhra Pradesh | 48.8 | 46.5 | -2.3 | 34.5 | 35.1 | 0.6 | |
| Arunachal Pradesh | 47.4 | 51.3 | 3.9 | 27.9 | 30.1 | 2.3 | |
| Assam | 52.4 | 51.9 | -0.5 | 37.7 | 38.6 | 0.9 | |
| Bihar | 66 | 61.6 | -4.5 | 55.8 | 52.2 | -3.6*** | |
| Chandigarh |  |  |  | 25.6 | 36.8 | 11.3 | |
| Chhattisgarh | 56.9 | 58.4 | 1.6 | 38.5 | 41 | 2.5 | |
| D&N Haveli |  |  |  | 33 | 39.5 | 6.6 | |
| Daman & Diu |  |  |  | 24 | 28.6 | 4.6 | |
| Delhi | 40.8 | 46 | 5.2 | 33.4 | 32.8 | -0.6 | |
| Goa | 28.1 | 25.7 | -2.3 | 29.3 | 14.7 | -14.7* | |
| Gujarat | 56.9 | 55.8 | -1.1 | 44.3 | 40.2 | -4.2* | |
| Haryana | 49.5 | 48.5 | -1 | 34.4 | 35.6 | 1.2 | |
| Himachal Pradesh | 43.1 | 47.3 | 4.2 | 29 | 30.4 | 1.4 | |
| Jammu & Kashmir | 42.8 | 35.4 | -7.3 | 31.3 | 29.8 | -1.5 | |
| Jharkhand | 51.6 | 55.5 | 3.9 | 49.8 | 48.4 | -1.4 | |
| Karnataka | 46.4 | 46.2 | -0.2 | 34.4 | 36.5 | 2.1 | |
| Kerala | 22.7 | 25.4 | 2.7 | 19 | 18 | -1 | |
| Lakshadweep |  |  |  | 36.8 | 24 | -12.8* | |
| Madhya Pradesh | 56.1 | 53.2 | -2.8 | 46.9 | 46.2 | -0.7 | |
| Maharashtra | 51.1 | 52.1 | 0.9 | 38 | 36 | -2 | |
| Manipur | 44.4 | 42.9 | -1.6 | 31.9 | 32.1 | 0.2 | |
| Meghalaya | 58.8 | 60.4 | 1.6 | 49.3 | 53.3 | 3.9 | |
| Mizoram | 44.1 | 48.8 | 4.7 | 35 | 33.7 | -1.4 | |
| Nagaland | 45.5 | 47.1 | 1.6 | 31.7 | 37.5 | 5.78** | |
| Orissa | 51.5 | 45 | -6.5* | 36 | 35 | -1.1 | |
| Pondicherry |  |  |  | 23.3 | 31 | 7.6 | |
| Punjab | 39.7 | 38.8 | -1 | 25.7 | 27.7 | 2 | |
| Rajasthan | 48.9 | 49.6 | 0.8 | 40.4 | 43.1 | 2.7* | |
| Sikkim | 42 | 46.4 | 4.4 | 28 | 36.1 | 8.2* | |
| Tamil Nadu | 31.6 | 35.7 | 4.2 | 25.2 | 25.8 | 0.6 | |
| Tripura | 40.6 | 38.9 | -1.7 | 25.8 | 29.2 | 3.4 | |
| Uttar Pradesh | 65.7 | 62.5 | -3.3 | 52.9 | 50.6 | -2.3** | |
| Uttaranchal | 45.9 | 52.9 | 7 | 35.4 | 33.2 | -2.3 | |
| West Bengal | 49.3 | 47.4 | -2 | 35.5 | 32.9 | -2.6 | |
| **India** | **53.9** | **52.4** | **-1.5** | **42.0** | **41.0** | **-1.0**** | |

*p<0.05, **p<0.01, ***p<0.001
